# The HHEX-ABI2/SLC17A9 axis induces cancer stem cell-like properties and tumorigenesis in HCC

**DOI:** 10.1186/s12967-024-05324-2

**Published:** 2024-06-06

**Authors:** Huizi Li, Jin Liu, Jie Lai, Xinyao Su, Xiaofeng Wang, Jiaqing Cao, Shengxun Mao, Tong Zhang, Qiuping Gu

**Affiliations:** 1 Department of General Surgery, The Second Affiliated Hospital of Nanchang University, Nanchang, 330006 Jiangxi China; 2 Department of Radiology, University of California, San Diego, The, USA; 3 Department of Gastrointestinal Surgery, The First Affiliated Hospital, Sun Yat-sen University, Guangzhou, Guangdong China; 4 Department of Hepatic Surgery and Liver Transplantation Center, The Third Actuated Hospital of Sun Yat-sen University, Guangzhou, 510000 Guangdong China; 5 Department of General Surgery, School of Medicine, Organ Transplantation Clinical Medical Center of Xiamen University, Xiang’an Hospital of Xiamen University, Xiamen University, Xiamen, 361000 China; 6 Department of Gastroenterology, Ganzhou People’s Hospital, No. 16, Meiguan Avenue, Zhanggong District, Ganzhou City, 341000 Jiangxi Province People’s Republic of China; 7 Department of Organ Transplantation, School of Medicine, Organ Transplantation Clinical Medical Center of Xiamen University, Xiang’an Hospital of Xiamen University, Xiamen University, Xiamen, 361005 Fujian China

**Keywords:** Hepatocellular carcinoma, Cancer stem cells, Molecular targeting, Signal transduction pathways, Translational oncology

## Abstract

Accumulating evidence indicated that HHEX participated in the initiation and development of several cancers, but the potential roles and mechanisms of HHEX in hepatocellular carcinoma (HCC) were largely unclear. Cancer stem cells (CSCs) are responsible for cancer progression owing to their stemness characteristics. We reported that HHEX was a novel CSCs target for HCC. We found that HHEX was overexpressed in HCC tissues and high expression of HHEX was associated with poor survival. Subsequently, we found that HHEX promoted HCC cell proliferation, migration, and invasion. Moreover, bioinformatics analysis and experiments verified that HHEX promoted stem cell-like properties in HCC. Mechanistically, ABI2 serving as a co-activator of transcriptional factor HHEX upregulated SLC17A9 to promote HCC cancer stem cell-like properties and tumorigenesis. Collectively, the HHEX-mediated ABI2/SLC17A9 axis contributes to HCC growth and metastasis by maintaining the CSC population, suggesting that HHEX serves as a promising therapeutic target for HCC treatment.

**Supplementary Information:**

The online version contains supplementary material available at 10.1186/s12967-024-05324-2.

## Introduction

Primary liver cancer is a highly prevalent malignancy and stands as the third leading cause of cancer-related deaths [1]. It encompasses two primary types, hepatocellular carcinoma (HCC) and intrahepatic cholangiocarcinoma (ICC), with HCC representing approximately 80% of all cases [2]. Despite remarkable progress in the diagnosis and treatment of liver cancer in recent years, the 5-year survival from HCC in the UK still remains below 20% [3]. Moreover, the incidence and mortality rates of liver cancer continue to escalate steadily, posing a significant societal and medical burden [4, 5]. From 1990 to 2019, the number of incident cases of primary liver cancer worldwide rose by 43.11%, from 373,393 to 534,365, while deaths increased by 32.68%, from 365,213 to 484,584 [5]. A recent nationwide analysis involving the financial burden of HCC found that hospitalizations rose from 67,779 (0.18% of admissions) in 2011 to 84,580 (0.23%) in 2017 and the average charges per admission increased significantly from $58,406 in 2011 to $78,791 in 2017 (*P* < 0.05) [6]. A key reason for this is that the underlying molecular mechanisms driving its pathogenesis are still largely unknown. Consequently, there exists an imperative demand for unveiling the intricate molecular pathways and therapeutic targets for liver cancer.

Emerging evidence suggests that cancer stem cells (CSCs), a specific subset of cancer cells capable of self-renewal and differentiation, play a crucial role in the development, recurrence, and metastasis of HCC [7]. Due to its self-renewal capacity, CSCs generate identical copies of themselves, leading to an expansion of the CSC population within the tumor [8]. Furthermore, CSCs exhibit the capacity to differentiate into diverse cell types, contributing to the observed heterogeneity within HCC tumors [8]. Another remarkable feature of CSCs is their resistance to conventional therapeutic approaches such as chemotherapy and radiation [9]. This resistance is believed to arise from various mechanisms, including the upregulation of drug efflux pumps and increased expression of anti-apoptotic proteins [10]. Several signaling pathways, including Wnt/β-catenin, Notch, and Hedgehog, have been implicated in the maintenance and regulation of CSCs in HCC [11]. Consequently, elucidating the underlying molecular mechanisms and developing effective treatments targeting CSCs may lead to improved outcomes for liver cancer.

HHEX is a highly conserved transcription factor belonging to the homeobox protein family. It could interact with other transcription regulators or directly bind to specific DNA sequences to regulate gene expression [12, 13]. HHEX is involved in fundamental processes such as cell proliferation and cell differentiation in various tissues. Moreover, emerging studies have indicated its association with tumorigenesis, although its expression and molecular functions may vary among different types of tumors [14]. In certain cancers, such as colorectal cancer and cholangiocarcinoma, HHEX was significantly overexpressed and has been shown to promote tumor proliferation and metastasis [15, 16]. Conversely, in breast, lung, and thyroid cancer, HHEX exerted inhibitory effects on tumor progression [17–19]. Previous research has suggested that HHEX could potentially serve as a biomarker for HCC progression [20]. However, the specific roles and mechanisms by which HHEX involving in HCC CSCs remained largely unclear. Accordingly, understanding the implications of HHEX in HCC CSCs was critical for unraveling the underlying molecular mechanisms and developing targeted therapeutic strategies.

In our present study, we identified that HHEX was overexpressed in HCC tissues and high expression of HHEX was associated with poor survival. Subsequently, we found that HHEX promoted HCC cell proliferation, migration, invasion, and stemness characteristics. Mechanistically, ABI2 serving as a co-activator of transcriptional factor HHEX upregulated SLC17A9 to promote HCC cancer stem cell-like properties and tumorigenesis. Our findings provide novel insight into the molecular mechanism by which HHEX facilitated the development and progression of HCC. Understanding the functional role of HHEX and its downstream targets may pave the way for the development of targeted therapies aimed at inhibiting HCC progression and improving patient outcomes.

## Materials and methods

### Silico analysis

We extended our investigation to the Liver Hepatocellular Carcinoma (LIHC) datasets in The Cancer Genome Atlas (TCGA), assessing HHEX expression in both HCC and normal tissue samples. Furthermore, UALCAN and GEPIA were utilized to delineate the relationship between ABI2 expression and prognosis as well as clinicopathological characteristics in HCC [21, 22]. Gene Set Enrichment Analysis (GSEA) was used to clarify the relationship between HHEX and HCC cancer stem cells using TCGA data.

### HCC specimens

The paraffin-embedded tissue microarray of HCC was acquired from Shanghai Outdo Biotech Company in Shanghai, China. Written informed consent and clinical characteristic statistics were collected from all patients. All protocols obtained approval from the Medical Ethics Committee of Shanghai Outdo Biotech Company.

### Cell culture and treatment

HCC cell lines PLC/PRF/5 and Hep3B were obtained from the Shanghai Branch of the Chinese Academy of Sciences. These cells were cultured in Dulbecco’s modified Eagle medium supplemented with 10% fetal bovine serum, 100 U/ml penicillin, and 100 µg/ml streptomycin at 37 °C in a CO2 incubator. Cell proliferation was assessed through real-time imaging using the IncuCyte ZOOM system, as previously described [23]. For the foci formation assay, 1000 HCC cells were seeded onto 6-well plates and incubated for 10 days. After incubation, the resulting colonies were stained with 1% crystal violet dye and quantified.

### Constitution of overexpression and knockdown HCC cells

To generate cells overexpressing HHEX, we cloned the complete human HHEX cDNA into the pCDH-CMV-MCS-EF1-Puro vector (Addgene). For HHEX knockdown, we introduced a specific short hairpin RNA (shRNA) targeting HHEX into the pLKO vector (Addgene). Stably transduced cells were selected using puromycin (Sigma-Aldrich). Target sequence against HHEX shRNA can be found in Supplementary Table S1.

### Flow cytometry

The CD133 antibody conjugated with APC was obtained from Miltenyi. The CD24 antibody conjugated with PE was procured from BD Biosciences. The CD326 antibody conjugated with FITC was purchased from STEMCELL. Flow cytometry analysis was conducted following the manufacturer’s instructions.

### Cell invasion and sphere formation assays

The cell invasion assay used a Transwell system, with cells pretreated with 0.5 µg/ml mitomycin C for 24 h to inhibit proliferation. In the upper chamber, 1 × 10^5 cells in 200 µl of DMEM were placed, while the lower chamber contained 500 µl of DMEM with 20% FBS as a chemoattractant. The setup incubated at 37 °C for 48 h facilitated cell invasion. Post-invasion, cells were fixed with paraformaldehyde for 10 min, non-invaders were removed, and the membrane was rinsed with PBS. Invading cells were then stained with 0.1% crystal violet for 10 min for microscopic examination. For the foci formation assay, a single-cell suspension in complete DMEM was prepared, with 1000 cells from various groups seeded in six-well plates and cultured for 2–3 weeks. Cells were then fixed with 4% paraformaldehyde for 20 min, stained with 0.5% crystal violet for 10 min, and colonies with 50 or more cells were counted. In sphere formation assays, cells were resuspended in DMEM containing bFGF(20 ng/ml), EGF (20 ng/ml), 1x B27, and 1x N2, and cultured in ultralow attachment 12-well plates at a density of 1000 cells per well.

### RT-qPCR

Total RNA was extracted using TRIzol reagent, and cDNA synthesis was performed with the Prime Script RT-PCR Kit (Takara, Japan). Real-time PCR assays were conducted following the manufacturer’s instructions using PowerUp SYBR Green Master Mix (Applied Biosystems, USA). The housekeeping gene GAPDH was used as a reference transcript for normalization. The relative mRNA expression levels of the target genes were analyzed using the 2^(-ΔΔCT) method. Detailed primer sequences can be found in Table S2.

### Western blot

Liver cancer cells were isolated by centrifugation and lysed in Radio Immunoprecipitation Assay (RIPA) buffer enhanced with a protease inhibitor cocktail. The protein concentrations in these lysates were quantified using the bicinchoninic acid (BCA) method. Proteins were then separated by electrophoresis on a 10% SDS-PAGE gel and transferred to a nitrocellulose membrane. To prevent non-specific binding, the membrane was blocked using 5% nonfat milk before being incubated with targeted primary antibodies at room temperature for one hour. Subsequent to this incubation, an HRP-conjugated secondary antibody was applied. Protein bands were detected using enhanced chemiluminescence (ECL) with a Genebox imaging system from Gene Company Limited (Hong Kong, China). The specifics of the antibodies used are detailed in Supplementary Table S3.

### Confocal assays

Confocal microscopy was employed to determine the subcellular distribution of HHEX and ABI2 within HCC cells. Cells were plated on glass coverslips and stabilized using a methanol fixative to preserve their structure. Primary antibodies specific to HHEX and ABI2 were applied, followed by detection with FITC-conjugated mouse monoclonal secondary antibodies for HHEX and PE-conjugated rabbit polyclonal antibodies for ABI2. Nuclei were stained using DAPI.

### Co-immunoprecipitation (Co-IP)

Cell lysates from HCC cells transfected with various vectors were used for immunoprecipitation, adhering to the guidelines provided by the manufacturer. This process was conducted at 4 °C overnight, employing 2–5 µg of specific antibodies for the capture. To ensure specificity, negative controls using IgG were included to account for non-specific interactions. The antibody-bound complexes were then incubated with protein A/G agarose beads at 4 °C. Following six washes in ice-cold lysis buffer, the isolated proteins were reconstituted in PBS and analyzed via SDS-PAGE, leading to further Western blot examination. The primer sequences for SLC17A9 used in ChIP assays are listed in Supplementary Table S4.

### RNA sequencing

Total RNA was isolated from PLC/PRF/5 cells transfected with shHHEX and shCon for subsequent RNA sequencing analysis (*GENOME*, Beijing, CN). The purity of the sample was determined by NanoPhotometer (IMPLEN, CA, USA). The concentration and integrity of RNA samples were detected by Agilent 2100RNA nano 6000 assay kit (Agilent Technologies, CA, USA). A total amount of 1–3 µg RNA per sample was used as input material for the RNA sample preparations. Sequencing libraries were generated using VAHTS Universal V6 RNA-seq Library Prep Kit for Illumina ® (NR604-01/02) following the manufacturer’s recommendations and index codes were added to attribute sequences to each sample. The clustering of the index-coded samples was performed on a cBot cluster generation system using HiSeq PE Cluster Kit v4-cBot-HS (Illumina) according to the manufacturer’s instructions. After cluster generation, the libraries were sequenced on an Illumina platform and 150 bp paired-end reads were generated. The cluster generation and sequencing were performed on Novaseq 6000 S4 platform, using NovaSeq 6000 S4 Reagent kit V1.5.

### Animal experiments

Four to six-week-old male NOD-SCID mice were procured from the Shanghai Model Organisms Center. These mice were categorized into three experimental groups for the tumor formation study: a control group, an HHEX knockdown group, and a group with both HHEX knockdown and SLC17A9 overexpression. Each group was administered subcutaneous injections containing either 50,000 or 20,000 HCC cells in the flank area. Over a 12-week observation period, tumor growth was carefully tracked using established protocols, and instances where no tumor developed were noted as negative outcomes. Additionally, male BALB/c nude mice, five weeks old, were also sourced from the same center. These mice received injections of 100 µl of PBS with HCC cells engineered for HHEX knockdown and SLC17A9 overexpression. Tumor sizes were determined using the formula: volume (mm³) = length × width² × 0.5.

### Immunohistochemical staining

Immunohistochemical staining of the tissue sections was carried out by exposing them to an HHEX antibody from Abcam at 4 °C overnight. In the negative control group, normal goat serum was used instead of the primary antibody. Two skilled pathologists, who were not informed of the patients’ biochemical and clinical data, independently evaluated the stained tissue sections. Their assessment method adhered to established protocols previously described in the literature [24].

### Statistical analysis

The presented data represent the mean ± SD from multiple independent experiments with triplicate samples. Group comparisons were assessed using the Student’s t-test. Survival outcomes, including overall survival and progression-free survival, were evaluated using the Kaplan-Meier method, and differences were analyzed using the log-rank test. We all performed univariate and multivariate analyses to clarify the association between HHEX expression and clinicopathologic variables in HCC. All the data analysis was carried out using GraphPad Prism 7 software for statistical analysis. Additional statistical analyses were performed using the SPSS software package (version 22; Chicago, IL, USA).

## Results

### HHEX overexpression is associated with poor prognosis in HCC patients

**Fig. 1:**
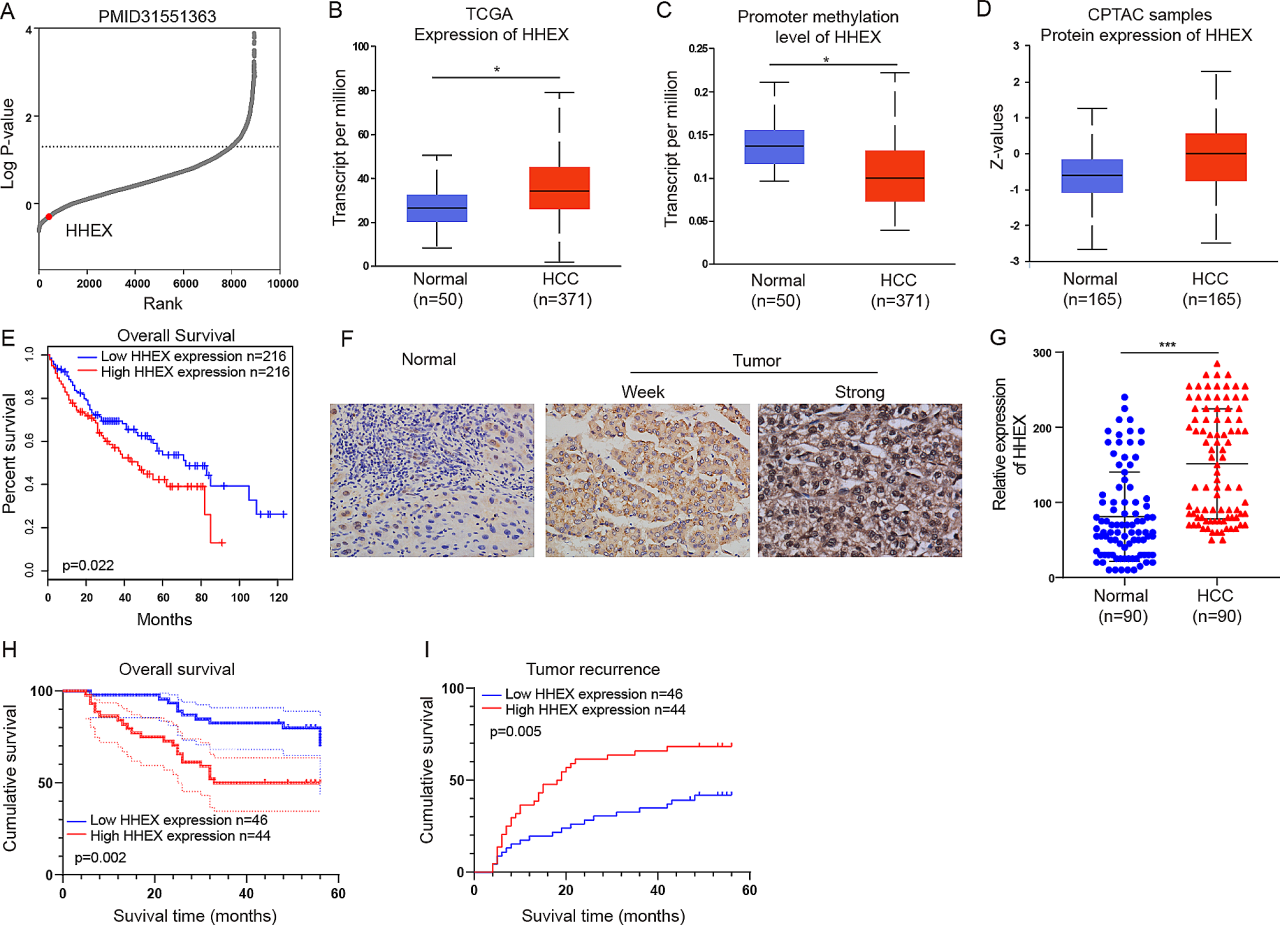
High HHEX expression predicts the poorer survival of HCC patients. (**A**) Genome-wide CRISPR knockout screening identified HHEX was the essential gene in hepatoma cell (PLC/PRF/F) proliferation. (**B**) The expression level of HHEX was higher in HCC tissues and normal liver tissues (UALCAN database). (**C**) The promoter methylation level of HHEX was lower in HCC tissues compared with Normal liver tissues (UALCAN database). (**D**) The protein expression level of HHEX was higher in HCC tissues than normal liver tissues based on CPTAC samples (UALCAN database). (**E**) Survival curves of patients with HCC showed that HHEX expression level was negatively correlated with prognosis prediction analyzed by GEPIA database. (**F**) The expression of HHEX in HCC and normal liver tissues were tested by IHC staining. (**G**) The protein expression level of HHEX was higher in HCC tissues than normal liver tissues in our tissues array. (**H**) Kaplan–Meier survival curve showed that HHEX expression level was negatively correlated with prognosis prediction of HCC analyzed by IHC in HCC tissue. (**I**) HHEX overexpression was positively correlated with tumor recurrence

An analysis in genome-wide CRISPR genome editing library screening for potential therapeutic targets for ATRX-mutated cancers indicated that HHEX was the top-ranking gene in hepatoma cell (PLC/PRF/F) proliferation, implying that HHEX is an essential gene in HCC progression (Fig. 1A). Through analyzing TCGA data using UALCAN, we identified that the mRNA level of HHEX was higher in HCC tissues and the promoter methylation level of HHEX was lower in HCC tissues as compared with normal liver tissues (Fig. 1B, C). Also, the protein expression level of HHEX was higher in HCC tissues than that in normal liver tissues using UALCAN database (Fig. 1D). Survival curves of patients with HCC showed that HHEX expression level was negatively correlated with prognosis analyzed by GEPIA database (Fig. 1E). Subsequently, we examined the protein expression of HHEX in our tissues array. The representative graph of weak, moderate and strong staining in our tissues array were shown in Fig. 1F. Western blot showed that the protein expression level of HHEX was higher in HCC tissues than normal liver tissues (Fig. 1G). Kaplan Meier survival curves demonstrated that high expression of HHEX was correlated with worse overall survival and higher recurrence rate in HCC patients (Fig. 1H, I). Furthermore, we explored the correlation of HHEX expressions with clinical pathologic characteristics in our tissues array. We found that HHEX overexpression was positively correlated with tumor size and tumor recurrence in chi-square analysis (Table 1). Consistently, HHEX overexpression also significantly correlated with tumor size and tumor recurrence in univariate and multivariate analyses (Table 2). Collectively, these results revealed that HHEX was highly expressed in HCC and high expression of HHEX was associated with worst prognosis in HCC patients.

**Table 1:** Correlation between the clinicopathologic variables and HHEX in HCC

Variables	Total	HHEX expression		P value
		Low expression	High expression	
Age(years)				
≤ 50	40	21	19	0.814
> 50	50	25	25	
Gender				
male	10	5	5	0.941
female	80	41	39	
Pathological Grades				
I	3	1	2	0.735
II	54	29	25	
III	33	16	17	
AJCC Grades				
I	63	33	30	0.343
II	25	13	12	
III	2	0	2	
Tumor size, cm				
≤ 5 cm	55	34	21	0.011*
> 5 cm	35	12	23	
Tumor number				
solitary	79	38	41	0.126
multiple	11	8	3	
Encapsulation				
complete	42	23	19	0.517
none	48	23	25	
Tumor recurrence				
no	41	27	14	0.010*
yes	49	19	30	
Cirrhosis				
no	9	5	4	0.779
yes	81	41	40	
Cirrhosis nodule				
solitary	9	4	5	0.673
multiple	81	42	39	
AFP				
< 20ug/L	36	17	19	0.547
≥ 20ug/L	54	29	25	
ALT				
normal	53	29	24	0.413
increase	37	17	20	
TB				
normal	76	40	36	0.501
increase	14	6	8	
GGT				
normal	44	24	20	0.524
decrease	46	22	24	
HBsAg				
negative	19	8	11	0.377
positive	71	38	33	
HBcAb				
negative	7	5	2	0.263
positive	83	41	42	

ALT, Alanine aminotransferase; HBsAg, hepatitis B surface antigen; HBcAb, hepatitis B core antigen AFP, alpha-fetoprotein; TB, total bilirubin; GGT, γ-glutamyl transpeptidase

**Table 2:** Univariate and multivariate analyses of clinicopathologic variables and HHEX in HCC

Variables	HHEX	HHEX	
	Univariate	Multivariate	
	P value	95%CI	P value
Age (≤ 50/>50y)	0.814		
Gender (male/female)	0.941		
Pathological Grades	0.896		
AJCC Grades	0.455		
Tumor size (≤ 5/>5 cm)	0.012*	1.102–6.824	0.03
Tumor number (solitary/multiple)	0.139		
Encapsulation (complete/none)	0.517		
Tumor recurrence (no/yes)	0.012*	1.109–6.576	0.029
Cirrhosis (no/yes)	0.779		
Cirrhosis nodule (solitary/multiple)	0.674		
AFP (< 20/≥20)	0.547		
ALT (normal/increase)	0.413		
TB (normal/increase)	0.503		
GGT (normal/decrease)	0.524		
HBsAg (negative/positive)	0.379		
HBcAb (negative/positive)	0.277		

CI, confidence interval; ALT, Alanine aminotransferase; HBsAg, hepatitis B surface antigen; HBcAb, hepatitis B core antigen AFP, alpha-fetoprotein; TB, total bilirubin; GGT, γ-glutamyl transpeptidase

### HHEX promotes HCC cell proliferation, migration and invasion

**Fig. 2:**
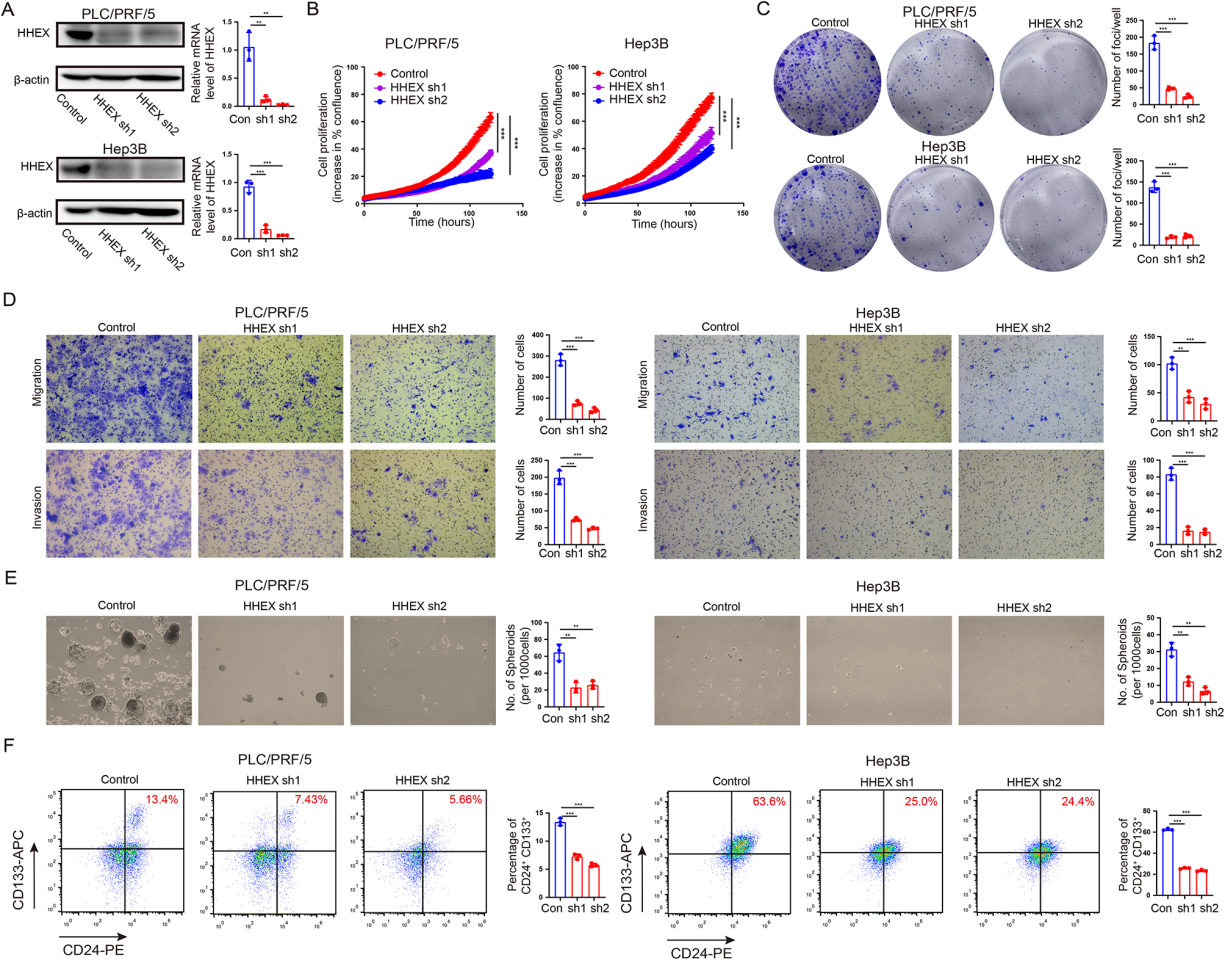
Knockdown of HHEX inhibits HCC cell proliferation, migration, invasion and cancer stem cells expansion. (**A**) shRNA against HHEX significantly suppressed the expression of HHEX in PLC/PRF/5 and Hep3B cells determined by western blotting and quantitative RT-PCR. Scrambled shRNA was used as negative control. (**B**) Summary graph showed the HCC cell 2D growth rate. (**C**) Foci formation assays were performed in PLC/PRF/5 and Hep3B cell lines to study tumor cell proliferation ability. D.E. Transwell assays were performed in PLC/PRF/5 and Hep3B cell lines to determine cell migration and invasion ability. E. Sphere formation assays were used to investigate HCC stemness characteristics in PLC/PRF/5 and Hep3B cells. F. Flow cytometric analysis of CD24^+^ CD133^+^ cell properties in PLC/PRF/5 and Hep3B cells

**Fig. 3:**
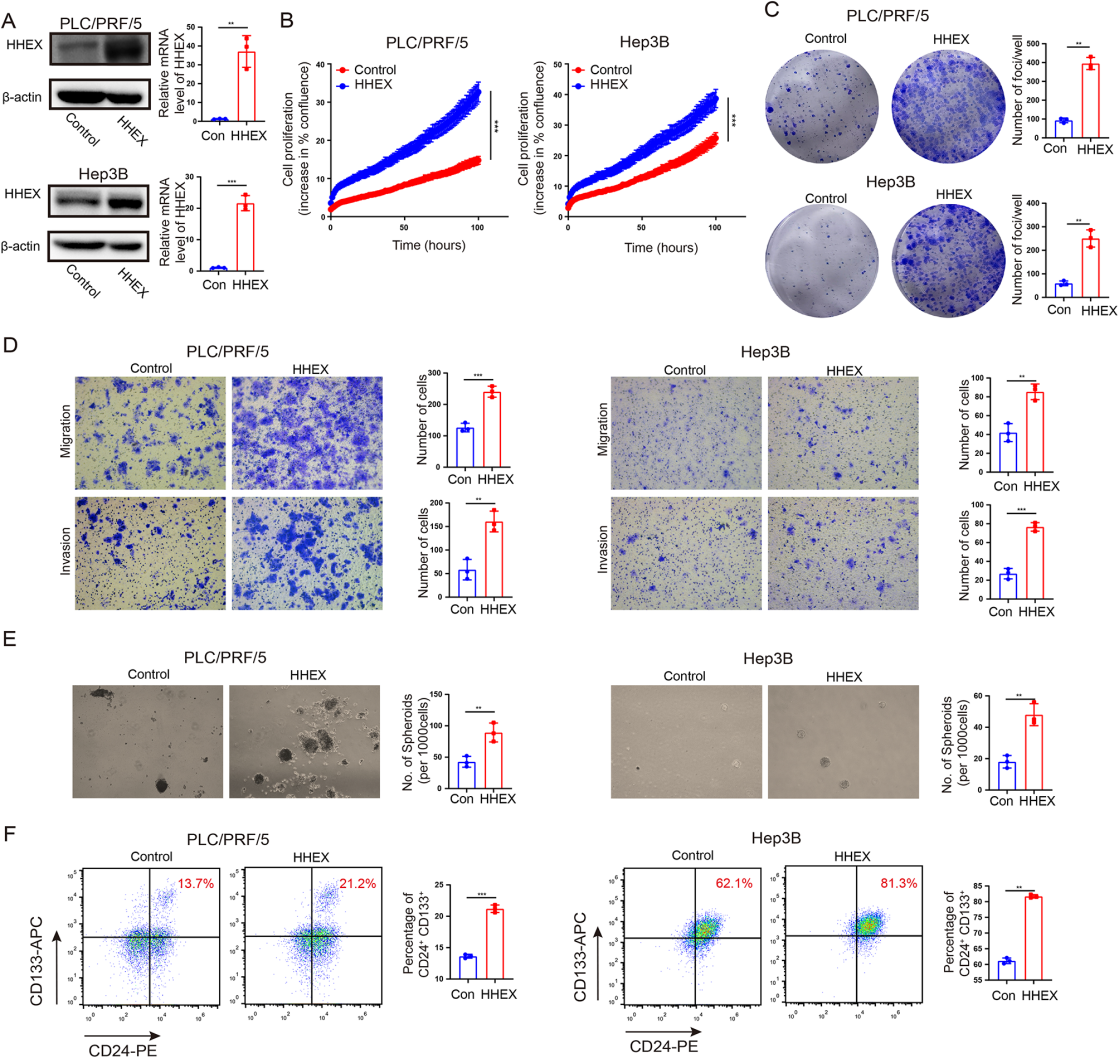
Ectopic expression of HHEX promotes cell proliferation, migration, invasion and stemness characteristics in HCC. (**A**) Western blot and qRT-PCR showed ectopic expression of HHEX in HHEX-transfected PLC/PRF/5 and Hep3B cells. (**B**) Summary graphs showed the ectopic expression of HHEX promoted HCC cell growth rate. (**C**) Foci formation frequency was significantly higher in HHEX-expressing cells compared to the control cells. (**D**) Overexpressing HHEX expression promoted cell migration and invasion in PLC/PRF/5 and Hep3B cells. (**E**) Sphere formation assays were used to investigate stemness characteristics in HHEX-expressing cells and control cells. (**F**) Flow cytometric analysis of CD24^+^ CD133^+^ cell properties in HHEX-expressing cells and control cells

The above expression and prognosis roles of HHEX implied that it may act as a potential oncogene in HCC. To further verify the possible functions of HHEX, we tried to clarify the effect of HHEX on HCC cell malignant biological behaviors through gain and loss of function experiments. Two cell lines (PLC/PRF/5 and Hep3B) were stably transfected with lentiviral shHHEX and scramble. The results of western blotting and qRT-PCR showed that shRNA against HHEX significantly suppressed the expression of HHEX in PLC/PRF/5 and Hep3B cells (Fig. 2A). Subsequently, 2D cell growth assays showed that knockdown of HHEX significantly suppressed HCC cell proliferation (Fig. 2B). Similarly, HHEX knocking down suppressed HCC cell growth in foci formation assays (Fig. 2C). Moreover, transwell assays indicated that knockdown of HHEX significantly inhibited cell migration and invasion ability in PLC/PRF/5 and Hep3B cell lines (Fig. 2D). Additionally, two cell lines (PLC/PRF/5 and Hep3B) were stably transfected with lentiviral HHEX overexpression and scramble. Western blot and qRT-PCR showed ectopic expression of HHEX in HHEX-transfected PLC/PRF/5 and Hep3B cells (Fig. 3A). The results of 2D cell growth assays and foci formation assays showed that ectopic expression of HHEX significantly promoted HCC proliferation (Fig. 3B, C). Furthermore, transwell assays demonstrated that overexpression of HHEX expression significantly facilitated cell migration and invasion in PLC/PRF/5 and Hep3B cells (Fig. 3D). Therefore, these results indicated that HHEX contributed to the HCC progression and metastasis, as evidenced by increased abilities in HCC cell growth, migration and invasion.

### HHEX promotes the stemness characteristics of HCC

Increasing evidence demonstrated that HCC CSCs could drive HCC initiation, metastasis, and recurrence [7, 25]. In our tissues array result, HHEX expression level was significantly associated with patient tumor recurrence. Accordingly, we tried to explore the role of HHEX in stem cell-like properties. Using TCGA data, GSEA indicated that high HHEX expression was positively associated with curated gene sets of stem cell in HCC (Fig. S1A-C). These results inferred that HHEX may be associated with stem cell-like properties in HCC. We examined the important role of HHEX in the stemness of HCC cells experimentally. Sphere formation assays indicated fewer and smaller spheroids in the shHHEX group than those in the control group (Fig. 2E), whereas more and bigger spheroids in the HHEX group than those in the control group (Fig. 3E). Moreover, CD24^+^CD133^+^ CSCs is a subset of HCC with highly tumorigenesis capacity and as less as 10 CD24^+^CD133^+^ cells could initiate tumor in NOD/SCID mice. Notably, knockdown of HHEX inhibited CD24^+^CD133^+^ CSCs population and overexpression of HHEX showed a conversely effect (Figs. 2F and 3F). Taken together, these results indicated that HHEX could promote the growth and metastasis of HCC via influencing HCC CSC properties.

### HHEX regulates SLC17A9 transcription via interaction with ABI2

**Fig. 4:**
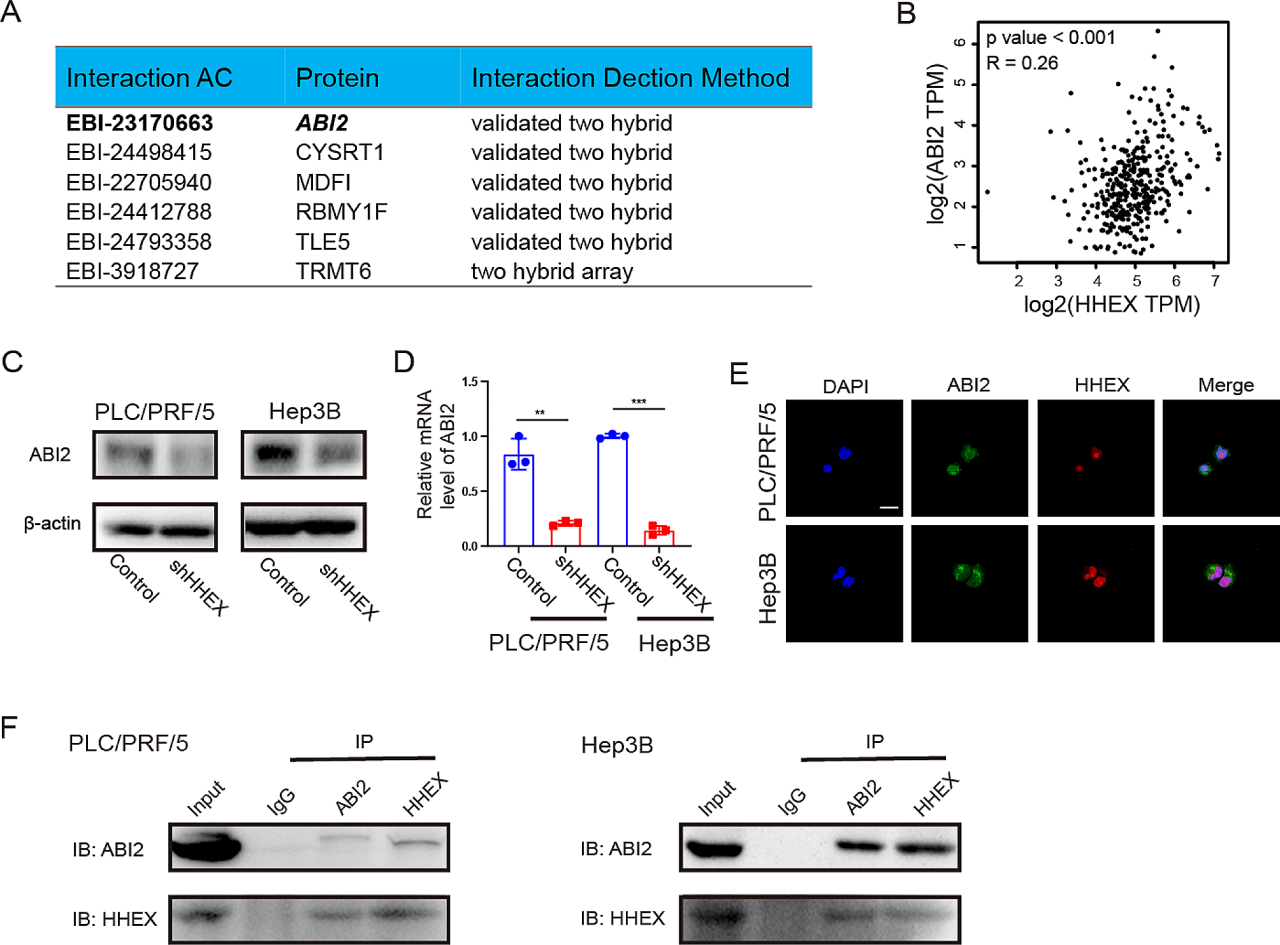
HHEX promotes and combines with ABI2 to promote HCC progression. (**A**) The datasheet showed HHEX interacting proteins from IntAct database. (**B**) Correlation analysis between the expressions of HHEX and ABI2 in HCC using GEPIA database. (**C**) D. Western blot and qRT-PCR showed knockdown of HHEX expression suppressed ABI2 expression level in HCC cell lines. E. IF double staining of HHEX and ABI2 in PLC/PRF/5 and Hep3B cells. Scale bar, 20 μm. F. Co-immunoprecipitation showed that HHEX directly bound to ABI2 in PLC/PRF/5 and Hep3B cells

Previous studies indicated that HHEX could interact with other transcription regulators to regulate downstream gene expression. Accordingly, to further clarify the potential mechanisms of HHEX triggering HCC progression, we tried to seek for the proteins directly binding to HHEX using IntAct database [26]. The datasheet showed that HHEX most likely bind to six proteins including ABI2, CYSRT1, MDF1, RBMY1F, TLE5, and TRMT6 (Fig. 4A). Interestingly, our previous study demonstrated that ABI2 acted as a co-activator of transcriptional factor MEOX2 upregulating KLF4-NANOG to promote liver cancer stem cell and tumour recurrence [23]. Correlation analysis using GEPIA database showed that ABI2 was positively correlated with HHEX in HCC patients (Fig. 4B). Moreover, western blot and qRT-PCR showed knockdown of HHEX expression suppressed ABI2 expression level in PLC/PRF/5 and Hep3B cells (Fig. 4C, D). Furthermore, we tried to verify whether HHEX interacted with ABI2 in HCC cells. Immunofluorescence showed that HHEX and ABI2 were co-localized in nucleus of PLC/PRF/5 and Hep3B cells (Fig. 4E). Furthermore, co-immunoprecipitation suggested that HHEX directly bound to ABI2 in PLC/PRF/5 and Hep3B cells (Fig. 4F). Taken together, these results indicated that HHEX directly bind to ABI2 and promoted the expression of ABI2.

**Fig. 5:**
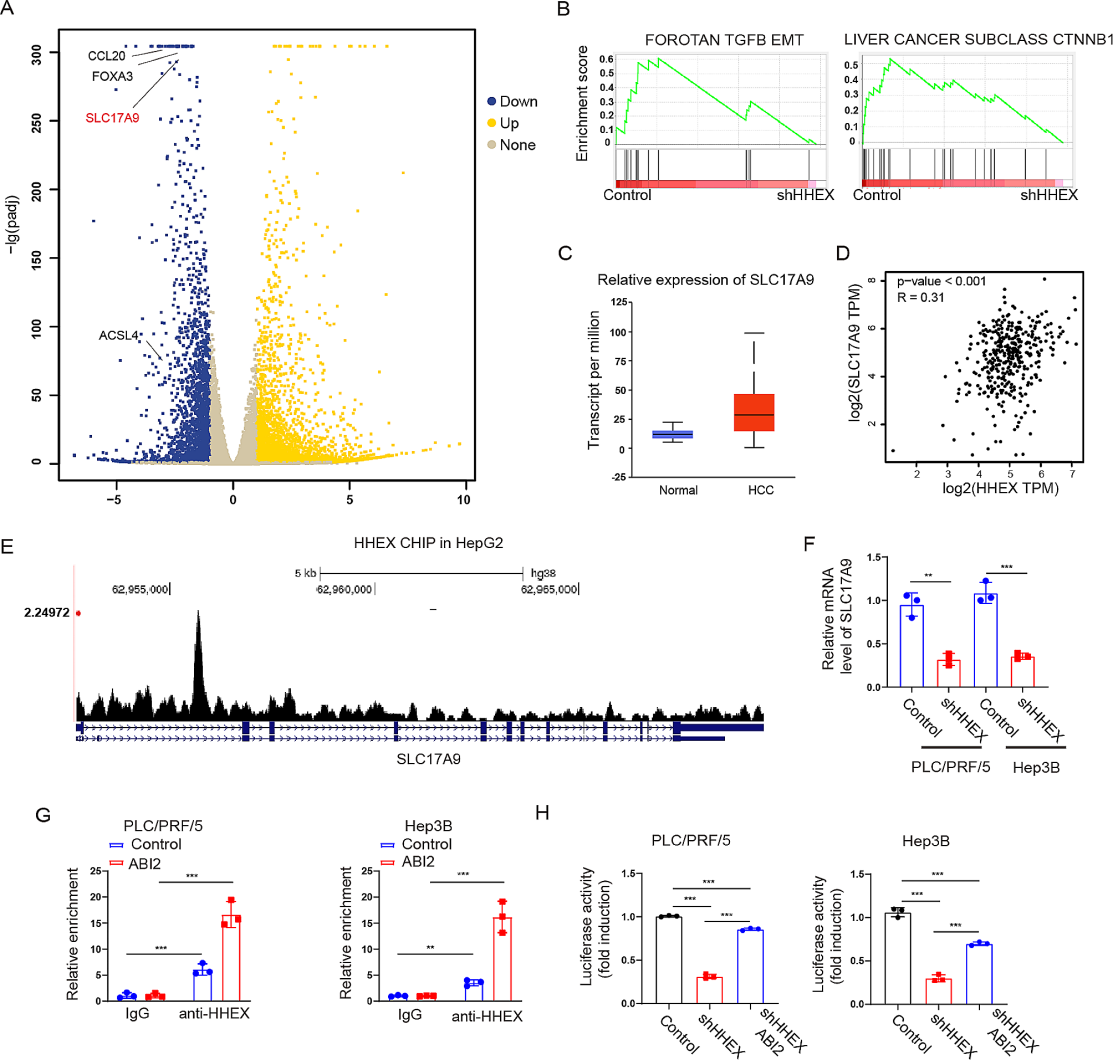
SLC17A9 is the performer in HHEX promoting HCC CSCs. (**A**) Volcano plots illustrate the distribution of the differential genes in the HHEX knockdown cell versus control cell. (**B**) Gene set enrichment analysis (GSEA) identified an enrichment of TGFB EMT and Liver cancer subclass CTNNB1. (**C**) The expressions level of SLC17A9 is higher in HCC tissues and normal liver tissues from UALCAN database. (**D**) Correlation analysis between the expressions of HHEX and SLC17A9 in HCC (GEPIA database). (**E**) HHEX CHIP-seq analysis in HepG2 cells using Cistrome Data Browser. (**F**) Knockdown of HHEX suppressed SLC17A9 expression. (**G**) HHEX binding to the promoters of SLC17A9 detected by ChIP-qPCR in PLC/PRF/5 and Hep3B cells. (**H**) PLC/PRF/5 and Hep3B cells were co-transfected with SLC17A9 promoter luciferase reporter and HHEX or vector plasmids followed by analysis of luciferase activity

To further uncover the downstream molecular mechanisms of HHEX, RNA sequencing was performed after HHEX knockdown in PLC/PRF/5 cells. A total of 6036 differentially-expressed genes were identified after HHEX knockdown (Fig. 5A). GSEA showed that these differentially-expressed genes mainly enriched in TGFB EMT and Liver cancer subclass CTNNB1 (Fig. 5B). In the Liver cancer subclass CTNNB1 enrichment subset, we found that SLC17A9 was the top-ranking gene and significantly suppressed after HHEX knockdown. Interestingly, a recent study found that SLC17A9 was significantly associated with Edmondson grade and distant metastasis and high expression of SLC17A9 was related to worst tumor-free survival and overall survival in HCC [27]. Consistently, we identified that the expressions level of SLC17A9 was higher in HCC tissues and normal liver tissues from UALCAN database (Fig. 5C). Correlation analysis showed that the mRNA expression level of HHEX was positively associated with SLC17A9 (Fig. 5D). Furthermore, Cistrome database identified that HHEX was bound to the promoter region of SLC17A9 in HepG2 cells [28], which suggested that HHEX may regulate SLC17A9 transcription (Fig. 5E). Inhibition of HHEX significantly suppressed the mRNA expression of SLC17A9 (Fig. 5F). Considering HHEX directly binding to ABI2, we wondered whether HHEX regulated SLC17A9 transcription via interaction with ABI2. Accordingly, ChIP-qPCR showed that HHEX bound to the promoter of SLC17A9, and overexpression of ABI2 enhanced this binding in PLC/PRF/5 and Hep3B cells (Fig. 5G). Moreover, luciferase reporter recomfired that knockdown of HHEX significantly inhibited the SLC17A9 promoter activity, which could be partly reversed by ABI2 overexpression in PLC/PRF/5 and Hep3B cells (Fig. 5H). Collectively, these findings indicated that HHEX regulates SLC17A9 transcription via interaction with ABI2.

### HHEX enhances HCC stemness characteristic through SLC17A9

**Fig. 6:**
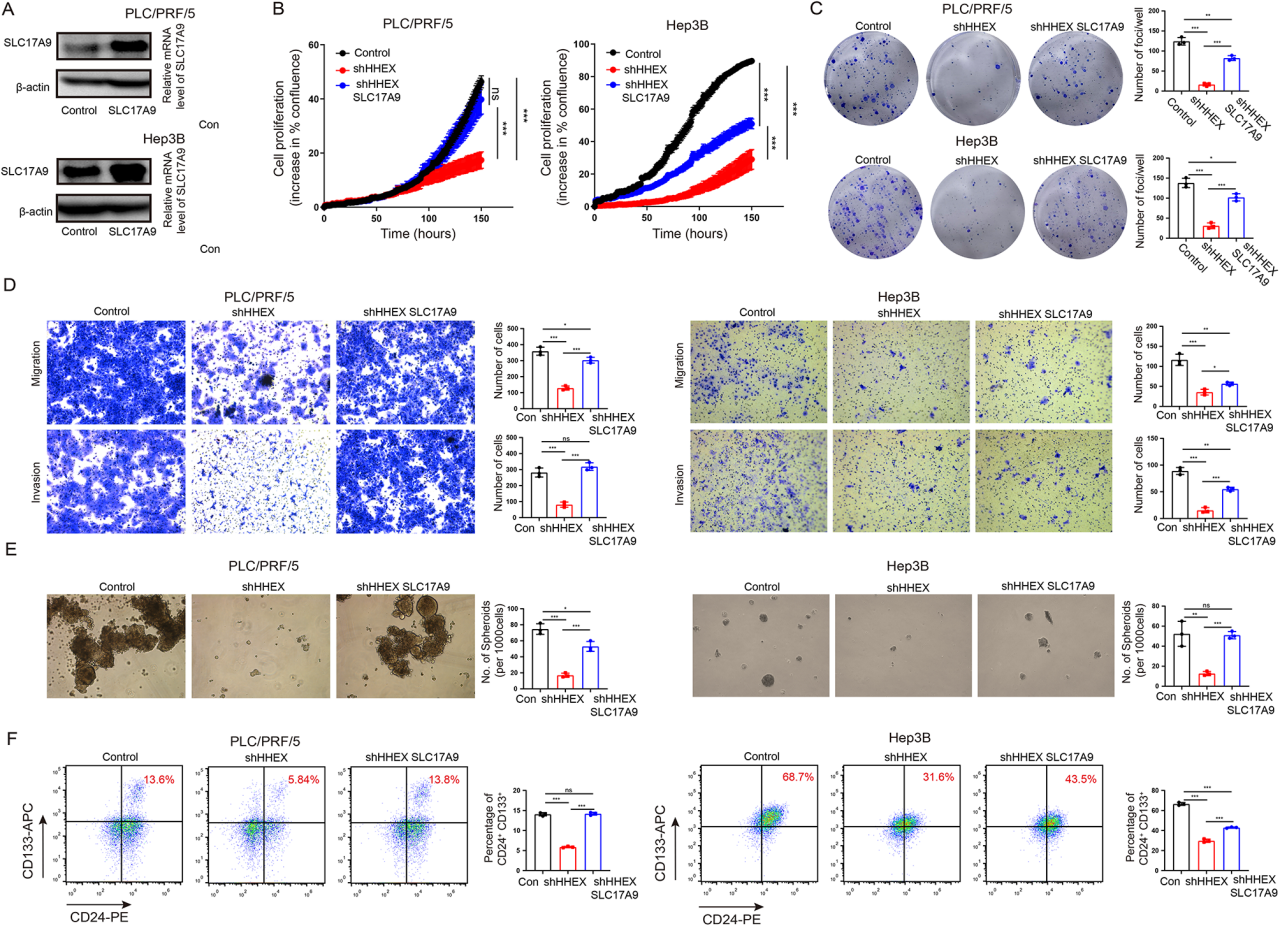
HHEX enhances HCC stemness characteristic through SLC17A9. (**A**) Western blot and qRT-PCR showing expression level of SLC17A9 in SLC17A9-transfected PLC/PRF/5 and Hep3B cells. (**B**) Summary graphs showed overexpressing SLC17A9 increase the growth rate of shHHEX cells. C.D. Overexpressing SLC17A9 effectively restored the foci formation, migration and invasion in shHHEX cells. E.F. Overexpressing SLC17A9 abrogated the stemness characteristic suppression effects in shHHEX cells

Furthermore, we tried to verify whether HHEX enhanced HCC stemness characteristic via SLC17A9. Western blot showed that the protein expression of SLC17A9 was significantly increased after overexpressing SLC17A9 (Fig. 6A). Cell growth assays and clone formation assay indicated that silencing HHEX significantly inhibited cell proliferation in PLC/PRF/5 and Hep3B cells, which could be partly mitigated by SLC17A9 overexpression (Fig. 6B, C). Transwell assays showed that knockdown of HHEX significantly inhibited cell migration and invasion in PLC/PRF/5 and Hep3B cells, which was partly restored by SLC17A9 overexpression (Fig. 6D). Sphere formation and flow cytometry assays revealed that inhibition of HHEX suppressed the CD24^+^ CD133^+^ cell populations and the formation volume in PLC/PRF/5 and Hep3B cells, which could be partly relieved by SLC17A9 overexpression (Fig. 6E, F). Collectively, these findings indicated that HHEX promotes HCC CSC properties through SLC17A9.

### HHEX promotes tumor growth, initiation and self-renewal in vivo

**Fig. 7:**
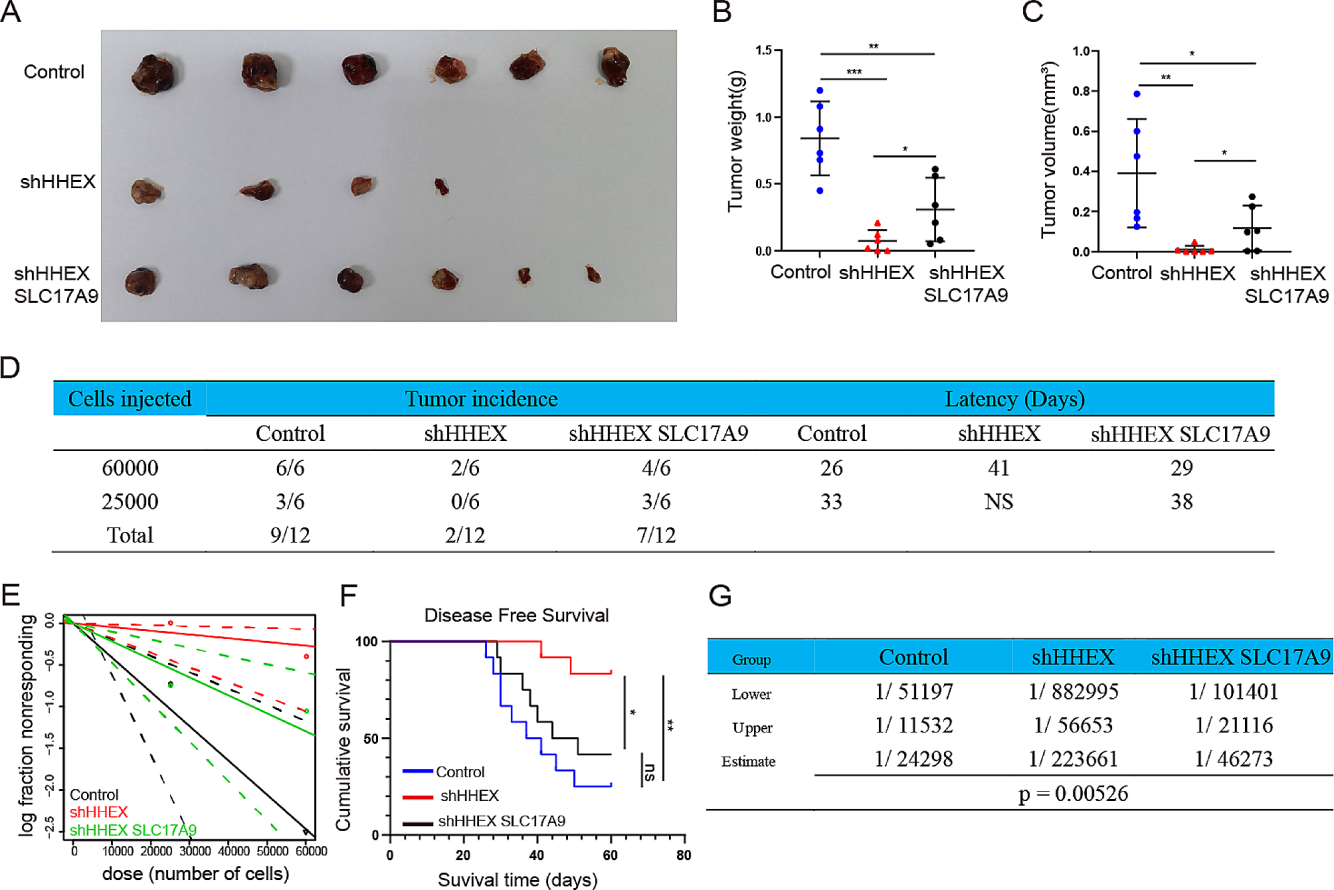
HHEX promotes HCC growth, initiation and self-renewal potential in vivo. **A-C**. Xenograft tumors induced by indicated cells in nude mice. Tumor weight and tumor volume of xenograft tumor were summarized. **D.G.** The indicated cells were inoculated subcutaneously in NOD-SCID mice. **E.** Extreme Limiting Dilution Analysis (ELDA) was used to calculated CSCs frequency. **F.** The disease-free survival curves of NOD-SCID mice

The above results indicated that HHEX promotes HCC cell proliferation, migration and invasion, and CSCs properties through SLC17A9 in vitro. We further clarify whether HHEX facilitated tumour growth, initiation and self-renewal potential through SLC17A9 in vivo. Xenografted tumour results showed that nude mice in shHHEX group had less tumour weight and tumour volume compared with those the control group, which could be partly reversed by SLC17A9 overexpression (Fig. 7A–C). Similarly, an in vivo LDA (limiting dilution assay) indicated that silencing of HHEX suppressed the tumorigenicity capacity and the CSCs frequency of HCC cells and improved NOD/SCID mouse disease-free survival, which could be partly restored by SLC17A9 overexpression (Fig. 7D–G). Collectively, HHEX promotes the CSCs population, improving HCC proliferation and tumorigenicity via SLC17A9.

## Discussion

HCC seriously threatens sufferers’ health and causes tremendous social and medical burden, but its potential pathogenesis still remains unclear. HHEX is a highly conserved transcription factor, which could bind to special DNA sequence or other transcription regulators to regulate gene expression. Previous studies revealed that HHEX played important roles in tumorigenesis, but the potential roles and potential mechanism of HHEX in HCC was largely unclear.

HHEX serves as an important transcription factor throughout liver development and is associated with liver bud formation and hepatoblast differentiation [29]. A previous study showed that there was a significant difference in HHEX expression between poorly differentiated HCC and well-differentiated HCC, but its possible roles in HCC were not further explored [20]. In the current study, we identified that HHEX may play a potential role in HCC progression through analyzing genome-wide CRISPR genome editing library. Moreover, we verified that HHEX was significantly upregulated in HCC tissues and high expression of HHEX was associated with unfavorable survival and clinicopathological characteristics, which indicated that HHEX may be a promising prognostic marker in HCC. Further findings revealed that overexpression of HHEX promoted HCC cells proliferation, migration and invasion, whereas inhibition of HHEX showed opposite effects. Increasing studies demonstrated that HHEX involved in regulated stem cell ontogeny. Jackson and coworkers found that HHEX regulated hematopoietic stem cell self-renewal and stress hematopoiesis through inhibiting Cdkn2a [30]. Kershaw and colleagues revealed that inhibition of HHEX promoted CSC-like properties of breast cancer cells [17]. Considering that CSCs were responsible for HCC tumor initiation and GSEA indicated that HHEX was positively linked with liver cancer stem cell, we further examined the effect of HHEX on HCC CSC properties. Our results showed that HHEX promoted the stemness characteristics of HCC, whereas inhibition of HHEX showed opposite effects. These findings revealed that HHEX exerted its pro-proliferation and pro-tumorigenicity effects by triggering stemness characteristics in HCC.

To explore the mechanism of HHEX promoting stemness characteristics in HCC, RNA-seq were performed to clarify the downstream target after silencing HHEX. SLC17A9 was identified as the top-ranking gene and enriched in Liver cancer subclass CTNNB1 enrichment subset. SLC17A9, a vesicular nucleotide transporter, mainly participated in cell transport and cell vitality, especially cancer cell ATP transport [31, 32]. Increasing studies found that high expression of SLC17A9 correlated with poor prognosis in some digestive system tumors, including gastric cancer [33], colorectal cancer [34], and HCC [27]. Kui and coworkers demonstrated that inhibition of SLC17A9 suppressed HepG2 cells proliferation, migration, and colony formation [35]. Li and colleagues revealed that SLC17A9 promoted proliferation and invasion of clear renal cell carcinoma through PTHLH [36]. A recent study identified that SLC17A9 may serve as a novel prognostic biomarker for osteosarcoma and SLC17A9 expression was associated with stemness in osteosarcoma [37]. These findings inferred that SLC17A9 may act as a potential oncogene and an independent risk factor for overall survival of patients with various tumors. In the current study, we showed that SLC17A9 was significantly overexpressed in HCC and high expression of SLC17A9 was linked with worst overall survival. Rescue experiments indicated that HHEX enhances HCC stemness characteristics, proliferation and invasion of HCC through SLC17A9 in vitro and in vivo. Previous studies showed that HHEX served as a transcription factor and our results found that HHEX was mainly located in nucleus of HCC cells. Subsequently, we found that HHEX was enriched in promotor region of SLC17A9 and ChIP-qPCR assays showed HHEX significantly bind with the promotor of SLC17A9. These findings revealed that HHEX was able to transcriptionally activate the expression of SLC17A9 in HCC.

Generally, gene expression regulation required the combined action of transcription factors and coactivator proteins [38, 39]. Also, previous studies showed that HHEX usually interacting with some coactivator proteins to mediate the transcription activity of the downstream genes. Guo and coworkers found that HHEX cooperated with YAP-TEAD4 complex to coregulate the expression of YAP/TEAD target genes, thus promoting colorectal tumorigenesis [15]. Studies have indicated that HHEX acted as a major transcriptional repressor by recruiting TLE corepressor proteins to target promoters to inhibit gene expression [40]. In the current study, we used IntAct database to predict that HHEX may interact with ABI2 to regulate SLC17A9 expression. Our previous study identified that ABI2 served as a co-activator of transcriptional factor MEOX2 upregulating KLF4/NANOG to promote liver cancer stem cell and drives tumor recurrence [23]. Interestingly, we found that HHEX and ABI2 could colocalize in the nucleus. Co-IP assays further demonstrated that HHEX could interact with ABI2 in HCC cells. ChIP-qPCR showed that more HHEX binding to the promoters of SLC17A9 and the enrichment effect was significantly enhanced after ABI2 overexpression in HCC cells. Moreover, luciferase reporter revealed that knockdown of HHEX significantly inhibited the SLC17A9 promoter activity, which could be partly reversed by ABI2 overexpression in HCC cells. These results indicated that HHEX interacted with ABI2 to upregulate the transcription activity of SLC17A9.

Our study still exists some potential limitations. Firstly, the findings were derived from a specific cohort of HCC tissue samples, which may limit the generalizability of the results. Future studies should validate the role of the HHEX-ABI2/SLC17A9 axis across a broader and more diverse set of samples to strengthen the applicability of these findings to varied patient populations. Secondly, while we propose that ABI2 acts as a co-activator of HHEX and upregulates SLC17A9, the precise molecular interactions and pathways remain partially elucidated. Detailed mechanistic studies are required to fully understand the interplay and regulatory mechanisms involved in this axis. Finally, while this study suggests that the HHEX-ABI2/SLC17A9 axis could be a potential therapeutic target, the direct applicability of these findings to clinical settings remains to be tested. Clinical trials are needed to establish the therapeutic utility and potential side effects of targeting this axis in HCC patients.

Collectively, we reported that HHEX was overexpressed in HCC tissues and significantly associated with tumor recurrence. Mechanically, HHEX played an important role in promoting liver CSCs stemness characteristic by binding with ABI2 to transcriptionally activating SLC17A9 expression. Importantly, knockdown of HHEX could suppress CSCs properties and tumorigenesis capacity, which contributed to providing a novel therapeutic target in HCC treatment.

### Electronic supplementary material

Below is the link to the electronic supplementary material.

**Supplementary Material 1**: **Figure S1:** Single gene GSEA of HHEX in HCC using TCGA database. (A-C) GSEA assessment of the enrichment score profile of stemness gene set in the HHEX high and low groups.

**Supplementary Material 2**

## Data Availability

All other supporting the findings of this study are available from the corresponding author upon reasonable request. Data sharing is not applicable as no new data were created or analyzed in this article.
