# Supplementary figures and images for: The HHEX-ABI2/SLC17A9 axis induces cancer stem cell-like properties and tumorigenesis in HCC
Source: J Transl Med. 2024 Jun 6;22:537. doi: 10.1186/s12967-024-05324-2 (PMC11155165; doi:10.1186/s12967-024-05324-2)

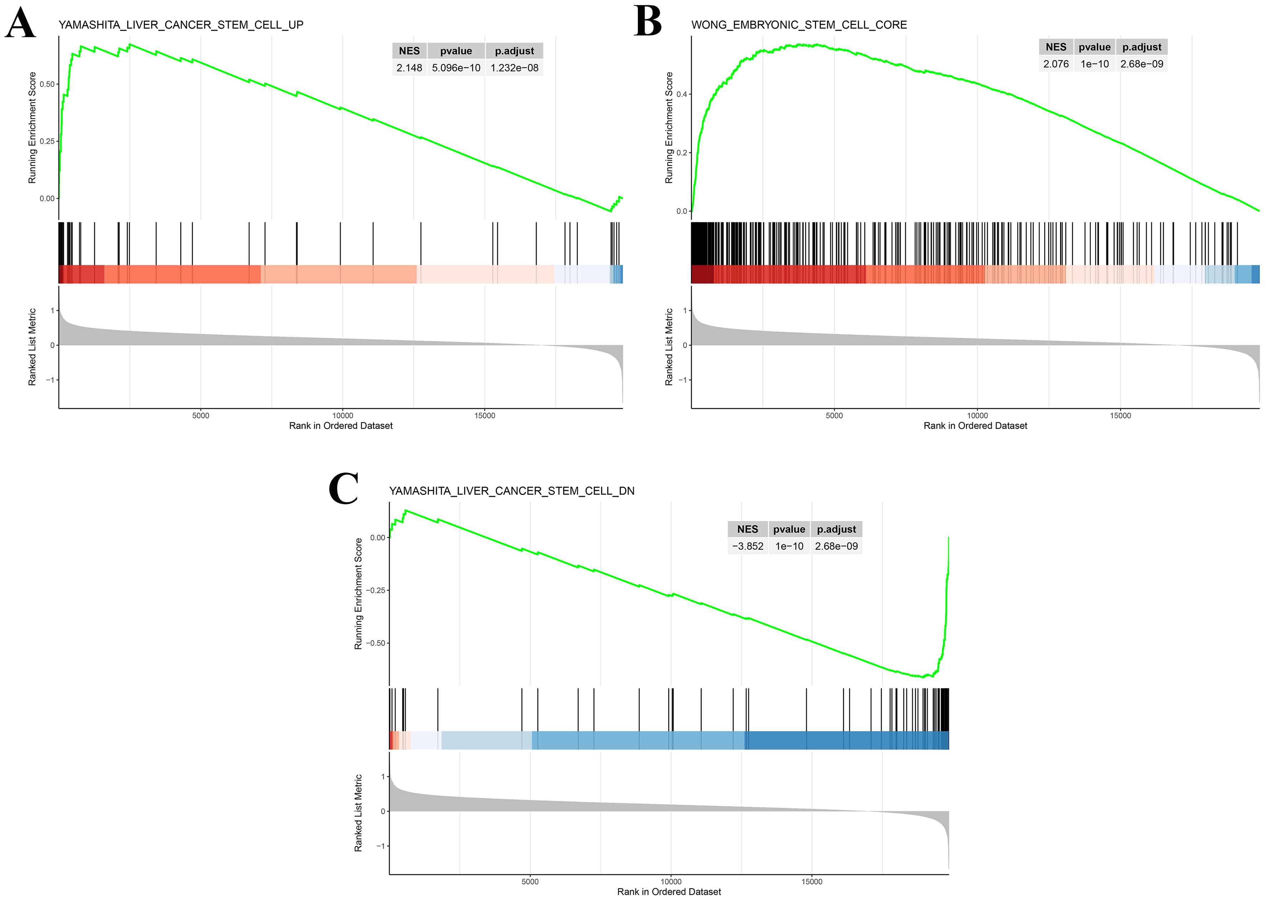

Supplement: Supplementary file 1 — Supplementary Material 1: Figure S1: Single gene GSEA of HHEX in HCC using TCGA database. (A-C) GSEA assessment of the enrichment score profile of stemness gene set in the HHEX high and low groups. [file 12967_2024_5324_MOESM1_ESM.tif]
