# Supplementary material for: The HHEX-ABI2/SLC17A9 axis induces cancer stem cell-like properties and tumorigenesis in HCC
Source: J Transl Med. 2024 Jun 6;22:537. doi: 10.1186/s12967-024-05324-2 (PMC11155165; doi:10.1186/s12967-024-05324-2)
Supplement: Supplementary file 2 — Supplementary Material 2 [file 12967_2024_5324_MOESM2_ESM.docx]

**Supplemental Table 1.** List of target sequence against HHEX shRNA

|  | \| Oligonucleotides \| (5' -> 3') \| \| --- \| --- \| |
| --- | --- | --- | --- |
| Target of shHHEX | CCCACTTAATGGAAAGGCAAA |

**Supplemental Table 2**. List of primer sequences used for qRT-PCR

|  | \|  \| Oligonucleotides (5' -> 3') \| \| --- \| --- \| |
| --- | --- | --- | --- |
| β-Actin Forward | CATGTACGTTGCTATCCAGGC |
| β-Actin Reverse | CTCCTTAATGTCACGCACGAT |
| HHEX Forward | ACGCCCTTTTACATCGAGGAC |
| HHEX Reverse | CGTGTAGTCGTTCACCGTC |
| ABI2 Forward | TGGCCGATTACTGCGAGAAC |
| ABI2 Reverse | GGGTGGTGTAGGCTTTGGTT |
| SLC17A9 Forward | CACCTCGGGGATCGGATTG |
| SLC17A9 Reverse | GCAGGGAAGTAAACCCCTTGG |

**Supplemental Table 3.** List of antibodies used in the study.

| Name | Catalog number | Clone number | Reference |
| --- | --- | --- | --- |
| Anti-HHEX  Anti-ABI2  Anti-SLC17A9 | cat. MAB83771  cat. ab108340  cat. NBP2-57119 | EPR3906 | 10.1038/s41556-022-01075-8  10.1038/oncsis.2014.3  10.1016/j.archoralbio.2019.104607 |
| PE-conjugated anti-CD24  APC-conjugated anti-CD133  Anti-β-Actin | cat. 555428  cat. 130-113-184  cat. AC004 | ML5  293C3 | 10.1186/bcr1610  10.1634/stemcells.2006-0258  10.1016/j.cell.2021.04.020 |

**Supplementary table 4.** primers sequences used for ChIP assays

| Name | Sequences 5’-3’ |
| --- | --- |
| SLC17A9 | Forward TAAGTGCAAGGCCCTGCCTTAGAC  Reverse TGCAGCCTTTCCCCAGGA |
